# Comparing Stent Thrombosis associated with Zotarolimus Eluting Stents versus Everolimus Eluting Stents at 1 year follow up: a systematic review and meta-analysis of 6 randomized controlled trials

**DOI:** 10.1186/s12872-017-0515-4

**Published:** 2017-03-16

**Authors:** Pravesh Kumar Bundhun, Chandra Mouli Yanamala, Wei-Qiang Huang

**Affiliations:** 1 Institute of Cardiovascular Diseases, The First Affiliated Hospital of Guangxi Medical University, Nanning, Guangxi 530021 People’s Republic of China; 2 Department of Internal Medicine, EALING Hospital, University of Buckingham, Uxbridge road, Southall, UB1 3HW London, UK

**Keywords:** Zotarolimus eluting stents, Everolimus eluting stents, Percutaneous coronary intervention, Definite stent thrombosis, Probable stent thrombosis, Drug eluting stents

## Abstract

**Background:**

Two thousand fifteen has been a winning year for Drug Eluting Stents (DES). Increase in the number of patients with cardiovascular diseases treated by Percutaneous Coronary Intervention (PCI) has resulted to a high demand for second generation DES. This current analysis aimed to compare the different types of Stent Thrombosis (ST) associated with Zotarolimus Eluting Stents (ZES) versus Everolimus Eluting Stents (EES) at 1 year follow up.

**Methods:**

Electronic databases were searched for studies comparing ZES with EES. Different types of ST reported at 1 year follow up were considered as the primary endpoints in this analysis. Odds Ratios (OR) with 95% Confidence Intervals (CIs) were used as the statistical parameters and the pooled analyses were carried out by the RevMan 5 · 3 software.

**Results:**

A total number of 10,512 patients were included in this analysis. No significant difference in any definite ST, acute definite ST, subacute definite ST, and late definite ST were observed between ZES and EES, at 1 year follow up with OR: 1.70, 95% CI: 0.92 – 3.16; *P* = 0.09, OR: 3.44, 95% CI: 0.82 – 14.43; *P* = 0.09, OR: 1.13, 95% CI: 0.43 – 2.95; *P* = 0.80 and OR: 2.39, 95% CI: 0.83 – 6.85; *P* = 0.11 respectively. Moreover, any definite or probable ST and definite/probable/possible ST were also not significantly different with OR: 1.39, 95% CI: 0.89 – 2.17; *P* = 0.15 and OR: 1.19, 95% CI: 0.84 – 1.70; *P* = 0.33 respectively. In addition, any probable ST, acute probable ST, late probable ST and possible ST were also not significantly different at 1 year follow up with OR: 1.11, 95% CI: 0.60 – 2.05; *P* = 0.75, OR: 0.53, 95% CI: 0.12 – 2.40; *P* = 0.41, OR: 1.67, 95% CI: 0.35 – 7.86; *P* = 0.52 and OR: 1.08, 95% CI: 0.64 – 1.82; *P* = 0.78 respectively.

**Conclusion:**

At 1 year follow up, ZES were not associated with significantly lower or higher definite and probable ST compared to EES. In addition, no significant difference was observed in acute, subacute and late definite or probable ST. However, further trials are recommended to assess the effects of these second-generation DES during the long-term.

## Background

Two thousand fifteen has been a winning year for Drug Eluting Stents (DES). Increase in the number of patients with cardiovascular diseases treated by Percutaneous Coronary Intervention (PCI) has resulted to a higher demand for second generation DES. Even if DES won the battle in terms of repeated revascularization when compared to Bare Metal Stents (BMS) [1], they also had short comings related mostly to long-term Stent Thrombosis (ST). Previously, several meta-analyses were carried out to compare ST associated with Sirolimus Eluting Stents (SES) and Paclitaxel Eluting Stents (PES), whereby SES were non-inferior to PES [2]. Later on, when Everolimus Eluting Stents (EES) were compared to non-EE DES, the formers were associated with a significantly lower rate of ST [3]. However, ST in patients treated with Zotarolimus Eluting Stents (ZES) and EES have seldom been analyzed using a large number of randomized patients. Previously published meta-analyses which focused mainly on the general adverse clinical outcomes associated with ZES and EES, did not specifically focus on the different types of ST following PCI [4, 5]. Hence, this current analysis aimed to compare ST associated with ZES versus EES at 1 year follow up, using a large number of patients extracted from randomized trials.

## Methods

### Data sources and search strategies

The Cochrane Library, MEDLINE or PubMed database of medical research articles, and EMBASE were searched by two authors (PKB and CMY), for English publications comparing ZES with EES using the words or phrase ‘zotarolimus eluting stents and everolimus eluting stents’. To widen this search strategy, the word ‘percutaneous coronary intervention’ and the abbreviations ‘ZES, EES and PCI’ were also used. Reference lists of suitable articles were also searched for relevant trials.

### Inclusion and exclusion criteria

Studies were included if:

- They were Randomized Controlled Trials (RCTs) which compared ZES with EES in patients who underwent PCI.
- They reported ST and other adverse outcomes as their clinical endpoints.
- They had a follow up period of 1 year.

Studies were excluded if:

- They were non-RCTs (observational studies, case studies, meta-analyses or letters to editors).
- They did not compare ZES with EES.
- They did not report ST or other adverse outcomes as their clinical endpoints.
- They were associated with the same trial.
- They were repeated trials or duplicates.

### Outcomes and follow up

The primary outcomes analyzed included ST defined by the Academic Research Consortium (ARC) [6]:

- Any definite ST;
- Acute definite ST;
- Subacute definite ST;
- Late definite ST;
- Any probable ST;
- Acute probable ST;
- Subacute probable ST;
- Late probable ST;
- Any definite or probable ST;
- Possible ST;
- Definite/probable or possible ST.

The secondary outcomes analyzed included:

- All-cause death;
- Cardiac death;
- Major Adverse Cardiac Events (MACEs) which were defined as a composite of all cause death, Myocardial Infarction (MI), emergent coronary artery bypass surgery (CABG) and clinically-indicated target lesion revascularization;
- Stroke;
- Patient-oriented composite endpoint consisting of all-cause mortality, MI and any coronary revascularization;
- MI;
- Target Vessel Revascularization (TVR);
- Target Lesion Revascularization (TLR);
- Target Vessel Failure (TVF);
- Target Lesion Failure (TLF).

These outcomes were followed for 1 year after PCI. Table 1 summarizes the primary outcomes reported in each trial whereas Table 2 lists the secondary outcomes with their corresponding follow up periods following PCI.

**Table 1:** Primary outcomes reported

Trial name	Primary outcomes and types of ST reported
DUTCH PEERS [17]	Any definite ST (0-360 d), acute definite ST (0-1 d), subacute definite ST (2-30 d), late definite ST (31-360 d), any definite or probable ST (0-360 d), any possible ST (0-360 d), any definite/probable/possible (0-360 d)
HOST-ASSURE [18]	Definite or probable ST, acute definite or probable ST, subacute definite or probable ST, early definite or probable ST, late definite or probable ST, any definite ST, acute definite ST, subacute definite ST, early definite ST, late definite ST, probable ST, acute probable ST, subacute probable ST, early probable ST, late probable ST, possible ST, acute possible ST, subacute possible ST, early possible ST, late possible ST
Lin 2015 [15]	ST
Mehilli 2013 [16]	Definite ST, probable ST
RESOLUTE [19]	Any definite ST (0-360 d), acute definite ST (0-1 d), subacute definite ST (2-30 d), late definite ST (31-360 d), any probable ST (0-360 d), acute probable ST (0-1 d), subacute probable ST (2-30 d), late probable ST (31-360 d), possible ST, definite or probable ST, definite/probable/possible ST
TWENTE [20]	Any definite ST (0-360 d), acute definite ST (0-1 d), subacute definite ST (2-30 d), late definite ST (31-360 d), any probable ST (0-360 d), acute probable ST (0-1 d), subacute probable ST (2-30 d), late probable ST (31-360 d), possible ST, definite or probable ST, definite/probable/possible ST

*Abbreviations*: *ST* stent thrombosis

**Table 2:** Secondary outcomes reported

Trial name	Other adverse outcomes	Follow up period	DAPT use
DUTCH PEERS [17]	TVF, all-cause death, cardiac death, TVMI, any TVR, clinically indicated TVR, clinically indicated TLR, TLF, MACEs, patient-oriented composite endpoint	12 months	1 year
HOST-ASSURE [18]	TLF, all-cause death, cardiac death, TVMI, repeated revascularization, TLR, TVR, CVA, TVF, patient-oriented composite endpoint	12 months	1 year
Lin 2015 [15]	Adverse cardiac events, all-cause death, cardiac death, MI	15 months	1 year
Mehilli 2013 [16]	All-cause death, MI, stroke, TLR, patient-oriented composite endpoint	12 months	1 year
RESOLUTE [19]	TLR, all-cause death, cardiac death, TVMI, clinically indicated TLR, MI, clinically indicated TVR, MACEs, patient-oriented composite endpoint, TVF	12 months	6 months to 1 year
TWENTE [20]	TVF, all-cause death, cardiac death, TVMI, clinically indicated TVR, TLF, clinically indicated TLR, MACEs, patient-oriented composite endpoint	12 months	1 year

*TVF* target vessel failure, *TVMI* target vessel related myocardial infarction, *TVR* target vessel revascularization, *TLF* target lesion failure, *MACEs* major adverse cardiac events, *CVA* cardiovascular accident, *TVF* target vessel failure, *MI* myocardial infarction, *DAPT* dual antiplatelet therapy

### Data extraction and review

The same two authors (PKB and CMY) who were involved in the search process, carefully reviewed the trials and assessed their methodological quality. The bias risk was assessed with reference to the Cochrane Collaboration [7]. The six components assessing the bias risk were taken into consideration and a score ranging from 0 to 2 was allocated to each component (low risk, unclear or high risk of bias). A maximum total score of 12 implied a very low risk of bias. The methodological information which were obtained from these trials were used to assess the bias risk, and was strictly dependent on what the authors have observed. Any feature which was missed during this assessment was ignored (an up and down of the score was possible). Grades were also allocated whereby a grade A implied a very low risk of bias whereas a grade E represented a very high risk of bias. Table 3 lists the scores and grades allocated to each eligible trial.

**Table 3:** Bias risk assessment according to the cochrane collaboration

Components assessed	DUTCH PEERS [17]	HOST-ASSURE [18]	Lin2015 [15]	Mehilli 2013 [16]	RESOLUTE [19]	TWENTE [20]
1	2	2	2	2	2	2
2	2	2	1	2	2	2
3	1	1	1	1	2	1
4	2	1	1	1	2	2
5	1	1	1	1	1	1
6	1	1	1	1	1	1
Total score	9	8	7	8	10	9
Bias grade	B	B	C	B	A	B

The six components recommended by the Cochrane Collaborations to assess bias risk

1: Sequence generation

2: Allocation sequence concealment

3: Blinding of participants and personnel

4: Blinding of outcome assessment

5: Incomplete outcome data

6: Selective outcome reporting and other potential bias

Moreover, information and data concerning the types of study reported, the patients’ enrollment period, the total number of patients treated by ZES and EES respectively, the reported primary and secondary outcomes, the follow up periods, the number of events that occurred in the study and the control groups, and information regarding the baseline features of the patients involved in this analysis were systematically extracted. Any disagreement or confusion concerning the eligibility of trials, or concerning the inclusion of certain data were discussed between these two authors, however, if a consensus could not be reached, disagreement was finally resolved by the third author (WQH).

### Statistical analysis

The Preferred Reporting Items for Systematic Reviews and Meta-Analyses [8] statement was considered relevant for this analysis which involved only randomized trials. Assessment of heterogeneity during the subgroup analysis was strictly dependent on the Cochrane Q-statistic test and the I^2^ statistic test. A *P* value of ≤ 0.05 was considered statistically significant. Moreover, an I^2^ value of 0% indicated no or very low heterogeneity, and an increasing percentage of I^2^ implied an increasing heterogeneity. In addition, a fixed effects model (I^2^ < 50%) and a random effects model (I^2^ > 50%) depending on the value of I^2^ obtained. Publication bias was assessed by visually observing funnel plots. Odds Ratios (OR) with 95% Confidence Intervals (CIs) were calculated and the subgroup analyses were carried out by the RevMan 5·3 software. All authors had full access to the trials and their data. Ethical or board review approval was not required for this type of research article.

## Results

### Search results

**Fig. 1:**
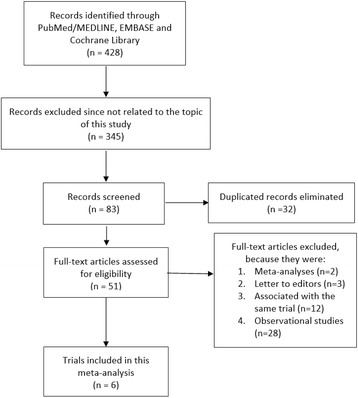
Flow diagram showing the study selection

Four hundred and twenty-eight (428) studies were identified from the electronic databases. Three hundred and forty-five studies were eliminated through abstracts and titles since they did not address any issue related to the idea of this research. A further 32 articles were eliminated since they replicated themselves. Fifty-one (51) full-text articles were assessed for eligibility. Seventeen (17) more articles were eliminated since they were meta-analyses (2), letters to editors (3) or they were associated with the same trial (12). Thirty-four (34) studies met most of the inclusion and exclusion criteria of this meta-analysis. However, since it involved only randomized trials, a further 28 studies were excluded because they were observational studies. Finally, 6 trials were included in this analysis (Fig. 1).

### General features of the trials included in this study

Table 4 shows the main features of the trials which were considered fully eligible for this analysis.

**Table 4:** General features of the trials included

Trials	Patients’ enrollment	Type of study	No of patients in ZES group (n)	No of patients in EES group (n)
DUTCH PEERS [17]	2010 - 2012	RCT	905	905
HOST-ASSURE [18]	2010 - 2011	RCT	1252	2503
Lin 2015 [15]	2008 - 2013	RCT	333	333
Mehilli 2013 [16]	2007 - 2011	RCT	324	326
RESOLUTE [19]	2008 - 2008	RCT	1121	1128
TWENTE [20]	2008 - 2010	RCT	695	692
Total no of patients (n)			4630	5887

*Abbreviations*: *ZES* zotarolimus eluting stents, *EES* everolimus eluting stents, *RCT* randomized controlled trials

A total number of 10,512 patients (4630 patients were treated by ZES and 5887 patients were treated by EES) were included in this analysis. Patients were enrolled from the year 2007 to the year 2013.

### Baseline features of the patients involved

The baseline features of the participants have been summarized in Table 5.

**Table 5:** Baseline features of the trials included

Trial name	Mean age	Males (%)	Ht (%)	Ds (%)	Cs (%)	DM (%)
	ZES/EES	ZES/EES	ZES/EES	ZES/EES	ZES/EES	ZES/EES
DUTCH PEERS [17]	64.0/65.0	73.0/73.0	55.0/53.0	46.0/48.0	24.0/26.0	18.0/17.0
HOST-ASSURE [18]	63.5/63.1	65.6/69.8	68.1/68.2	65.7/64.0	29.5/32.9	32.0/31.8
Lin 2015 [15]	63.0/65.5	72.8/73.4	71.9/69.6	59.3/57.4	64.8/51.4	25.5/25.2
Mehilli 2013 [16]	69.4/70.2	72.8/77.3	68.2/69.9	68.8/75.8	14.8/13.2	28.4/28.5
RESOLUTE [19]	64.4/64.2	76.7/77.2	71.1/71.3	63.9/67.7	26.5/26.5	23.5/23.4
TWENTE [20]	63.9/64.5	72.5/72.5	55.4/55.8	57.0/61.4	25.3/23.6	22.7/20.6

*Abbreviations*: *Ht* hypertension, *Ds* dyslipidemia, *Cs* current smoker, *DM* diabetes mellitus, *ZES* zotarolimus eluting stents, *EES* everolimus eluting stents

According to Table 5, no significant difference was observed in the baseline features among patients who were treated by ZES and EES respectively.

### Stent Thrombosis associated with ZES and EES at 1 year follow up

Results of this analysis has been summarized in Table 6.

**Table 6:** Results of this analysis

Outcomes analyzed	No of trials reporting these outcomes (n)	OR with 95% CI	*P* value	I^2^ (%)
Any definite ST	6	1.70 [0.92 – 3.16]	0.09	43
Acute definite ST	4	3.44 [0.82 – 14.43]	0.09	0
Subacute definite ST	4	1.13 [0.43 – 2.95]	0.80	46
Late definite ST	4	2.39 [0.83 – 6.85]	0.11	0
Any probable ST	6	1.11 [0.60 – 2.05]	0.75	20
Acute probable ST	3	0.53 [0.12 – 2.40]	0.41	0
Subacute probable ST	3	0.98 [0.14 – 6.63]	0.98	67
Late probable ST	3	1.67 [0.35 – 7.86]	0.52	0
Any definite or probable ST	6	1.39 [0.89 – 2.17]	0.15	7
Possible ST	4	1.08 [0.64 – 1.82]	0.78	0
Definite/probable or possible ST	3	1.19 [0.84 – 1.70]	0.33	0

*Abbreviations*: *OR* odds ratios, *CI* confidence intervals, *ST* stent thrombosis

**Fig. 2:**
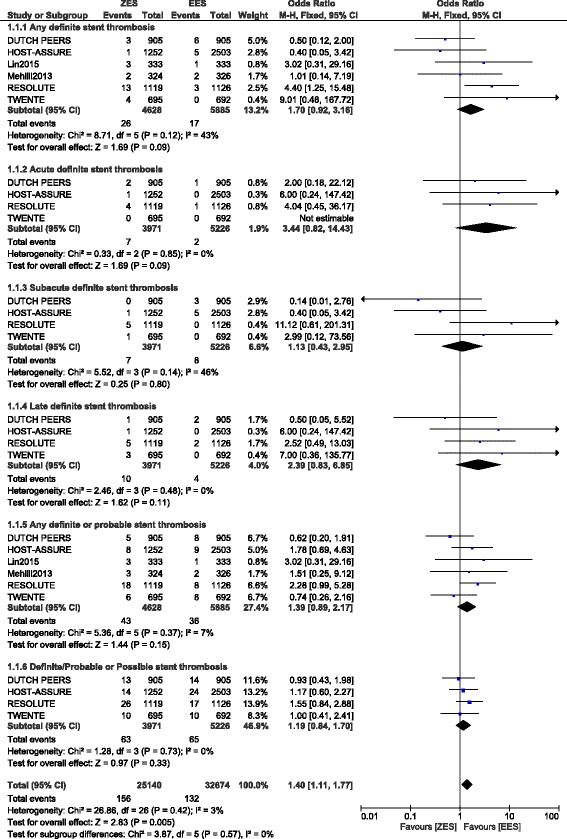
Types of Definite Stent Thrombosis associated with ZES versus EES

No significant difference was observed between ZES and EES when analyzing any definite ST, acute definite ST, subacute definite ST, and late definite ST observed at 1 year follow up with OR: 1.70, 95% CI: 0.92 – 3.16; *P* = 0.09, OR: 3.44, 95% CI: 0.82 – 14.43; *P* = 0.09, OR: 1.13, 95% CI: 0.43 – 2.95; *P* = 0.80 and OR: 2.39, 95% CI: 0.83 – 6.85; *P* = 0.11 respectively when a fixed effects model was used. Another analysis was carried out using a random effects model to analyze the subgroup ‘any definite ST’. However, similarly, no significant difference was observed with OR: 1.53, 95% CI: 0.56 – 4.17; *P* = 0.41. Moreover, any definite or probable ST and definite/probable/possible ST were also not significant between these two types of second-generation DES with OR: 1.39, 95% CI: 0.89 – 2.17; *P* = 0.15 and OR: 1.19, 95% CI: 0.84 – 1.70; *P* = 0.33 respectively. Results showing definite ST and its subtypes have been illustrated in Fig. 2.

**Fig. 3:**
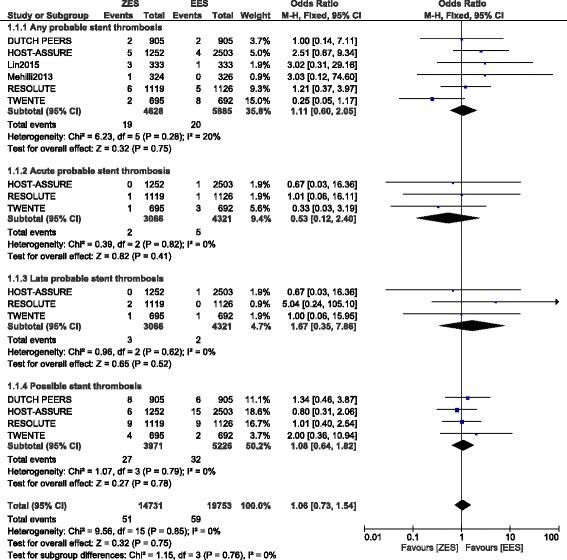
Types of Probable Stent Thrombosis associated with ZES versus EES

**Fig. 4:**
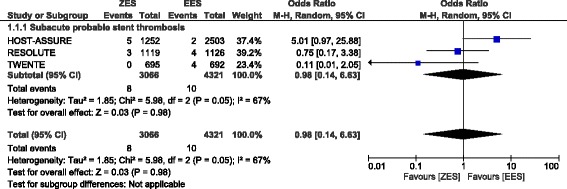
Subacute Probable Stent Thrombosis associated with ZES versus EES

Any probable ST, acute probable ST, late probable ST and possible ST were also not significantly different with OR: 1.11, 95% CI: 0.60 – 2.05; *P* = 0.75, OR: 0.53, 95% CI: 0.12 – 2.40; *P* = 0.41, OR: 1.67, 95% CI: 0.35 – 7.86; *P* = 0.52 and OR: 1.08, 95% CI: 0.64 – 1.82; *P* = 0.78 respectively at 1 year following PCI (Fig. 3). Since a high level of heterogeneity was observed when analyzing subacute probable ST, a random effects model was used to analyze this subgroup which showed comparable result between these two types of second-generation DES with OR: 0.98, 95% CI: 0.14 – 6.63; *P* = 0.98 (Fig. 4).

### Other adverse clinical outcomes associated with ZES and EES at 1 year follow up

**Fig. 5:**
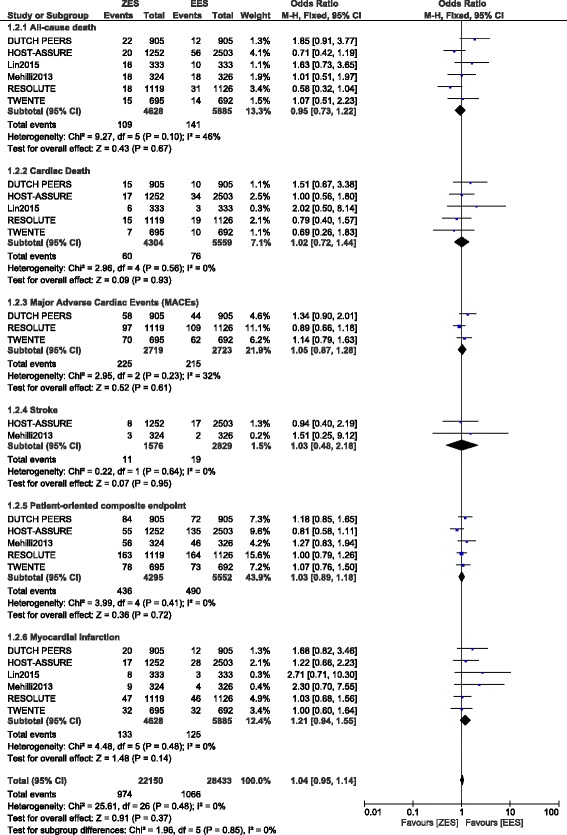
Adverse clinical outcomes associated with ZES versus EES

Although this analysis assessed the different types of ST manifested between ZES and EES, other adverse clinical outcomes were also analyzed. The current results showed a comparable rate of all-cause death and cardiac death between ZES and EES with OR: 0.95, 95% CI: 0.73 – 1.22; *P* = 0.67 and OR: 1.02, 95% CI: 0.72 – 1.44; *P* = 0.93 respectively. MACEs, stroke, patient-oriented composite endpoints, and MI were also similarly observed between ZES and EES with OR: 1.05, 95% CI: 0.87 – 1.28; *P* = 0.61, OR: 1.03, 95% CI: 0.48 – 2.18; *P* = 0.95, OR: 1.03, 95% CI: 0.89 – 1.18; *P* = 0.72 and OR: 1.21, 95% CI: 0.94 – 1.55; *P* = 0.14 respectively (Fig. 5).

**Fig. 6:**
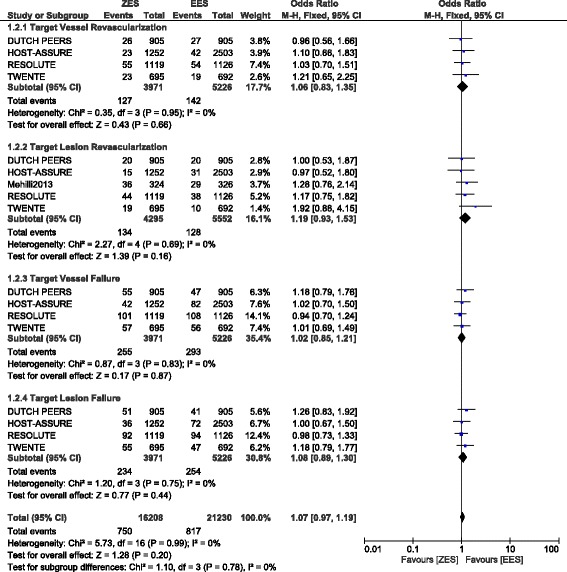
Repeated Revascularization associated with ZES versus EES

This analysis showed a similar rate of TVR, TLR, TVF and TLF reported between ZES and EES with OR: 1.06, 95% CI: 0.83 – 1.35; *P* = 0.66, OR: 1.19, 95% CI: 0.93 – 1.53; *P* = 0.16, OR: 1.02, 95% CI: 0.85 – 1.21; *P* = 0.87 and OR: 1.08, 95% CI: 0.89 – 1.30; *P* = 0.44 respectively (Fig. 6).

### Sensitivity analysis

Sensitivity analysis was also carried out to find out whether the results were influenced by any of the trial which was included during the subgroup analysis. Trials were excluded one at a time, and then a new analysis was carried out. This process was repeated with the exclusion of a different trial each time. Within the subgroup assessing ‘any definite ST’, excluding trial DUTCH PEERS and HOST ASSURE respectively, showed significant results supporting EES with OR: 2.44, 95% CI: 1.17 – 5.08; *P* = 0.02 and OR: 2.05, 95% CI: 1.04 – 4.04; *P* = 0.04 respectively. In addition, when trial DUTCH PEERS was excluded when analyzing late definite ST, a statistically significant result favoring EES was obtained with OR: 3.73, 95% CI: 1.02 – 13.59; *P* = 0.05. However, the significance approached the cut-off value. When the same trial was excluded while analyzing ‘any definite or probable ST’, the result again approached statistical significance with OR: 1.64, 95% CI: 1.00 – 2.68; *P* = 0.05. Nevertheless, consistent results were obtained throughout all the other subgroups. Excluding other trials did not show any significance compared to the main results obtained.

### Publication bias

**Fig. 7:**
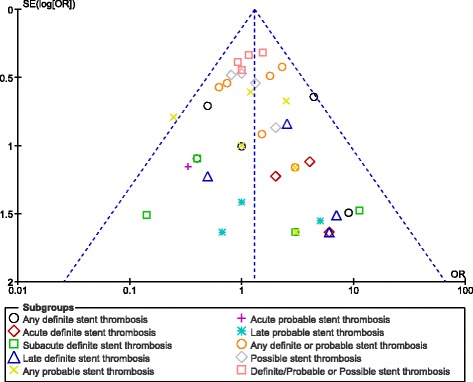
Funnel plots showing publication bias

**Fig. 8:**
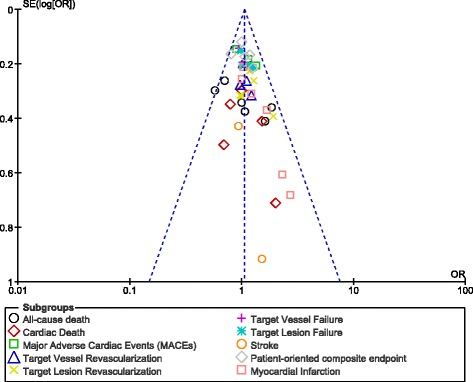
Funnel plots showing publication bias

After visually assessing the funnel plots, a low publication bias was observed among most of the subgroups analyzing the different subtypes of ST and other adverse clinical outcomes in these patients treated by ZES versus EES at 1 year follow up. The funnel plots representing publication bias have been illustrated in Figs. 7 and 8.

## Discussion

This analysis aimed to compare ST reported between ZES and EES in patients with coronary artery disease during a 1 year follow up. The current results showed no significant difference in the subgroup analyzing any definite or probable ST, acute definite or probable ST, subacute definite or probable ST, late definite or probable ST, possible ST and definite, probable or possible ST at 1 year follow up. Moreover, among the subgroups analyzing MACEs, mortality, MI, stroke, and repeated revascularization, no significant difference was observed between ZES and EES.

A meta-analysis [4] comparing the long-term effect of second generation DES for coronary artery disease, published by Li et al, showed ZES and EES to be associated with a similar efficacy and safety profile. EES did not reduce the rate of ST defined by the ARC with OR: 0.83, 95% CI: 0.56 – 0.25; *P* = 0.37. In contrast to their analysis, this current analysis involved different subgroups of ST with a larger number of randomized patients. Moreover, the study by Li et al was limited to the fact that only two studies reported data on very late ST. Another meta-analysis comparing the efficacy and safety of EES and ZES showed that among randomized trials, ZES and EES were comparable [5]. However, among data obtained from observational studies, EES were associated with significantly lower rates of ST and MACEs compared to ZES. When the data from randomized trials and observational studies (published and unpublished studies) were pooled, the results still showed EES to be associated with a significantly lower rate of ST compared to ZES. However, a random effects model was used during the analysis due to the presence of a high level of heterogeneity. Because the meta-analysis published by Gu et al showed a comparable rate of ST when randomized data were considered whereas ZES were associated with a significantly higher rate of ST compared to EES when data obtained from observational cohorts were used, future analysis should include either data obtained only from randomized trials, or patients obtained only from observational cohorts without combining them together.

The patient-related and stent-related outcomes from the multicenter prospective EXCELLENT and RESOLUTE-Korea Registries which were observational cohorts consisting of 5054 patients showed a similar rate of definite and probable ST with ZES and EES at 1 year follow up, which was also the case for this current analysis involving data obtained only from randomized trials [9].

Even if this current analysis had a follow up period of 1 year, the study published by Lee et al including Korean patients undergoing new-generation DES implantation had a follow up of 33 months (2.8 years) whereby comparable clinical outcomes were observed between ZES and EES [10]. A total number of 9 patients developed ST defined by ARC, however, no significant difference was observed between ZES and EES.

Moreover, results provided by the THCRIC registry also showed no significant difference in clinical outcomes between ZES and EES during a 1 year follow up after PCI [11]. However, ST were not reported in this observational study. Also, Omar et al showed comparable ST between EES and ZES; but, when EES were compared to SES, a lower rate of ST was observed in the EES group whereas ST between EES and BMS were also similar [12].

When randomized trials were compared, both ZES and EES were associated with similar rates of ST and other adverse clinical outcomes as reported in the DUTCH PEER trial, and both types of stents could be recommended in the general population with coronary artery diseases. The RESOLUTE and TWENTE trials also reported comparable ST between ZES and EES further supporting the results of this current analysis. At last, even data obtained from a German DES registry, showed first and second generation DES to be clinically equivalent at least at 1 year follow-up [13].

A recently published meta-analysis showed Dual Anti-Platelet Therapy (DAPT) use for less or equal to six months following PCI with EES or ZES not to cause any increase or decrease in ST [14]. Even though in this current analysis, almost all the patients were on dual antiplatelet therapy (DAPT) for 1 year, still no significant change in ST were observed. However, future trials will have to show the effect of a longer length of DAPT use on the occurrence of ST.

### Novelty

This study is new in the way that it involved a large number of randomized patients compared to previously published studies. By excluding patients obtained from observational cohorts, this analysis involved only good data which resulted in a low level of heterogeneity during the subgroup analysis. Moreover, previously published meta-analyses did not specifically focus on ST. This current study analyzed all possible subtypes of ST including any definite or probable ST, acute definite or probable ST, subacute definite or probable ST, late definite or probable ST, possible ST and definite/probable/possible ST. Furthermore, other adverse clinical outcomes were also analyzed in details. In addition, bias risk assessment was carried out, which was not the case in other previously published meta-analyses.

### Limitations

Similar to other studies, this systematic review and meta-analysis also had limitations. First of all, due to the limited number of patients analyzed, the results might be affected. In addition, one trial reported ST during a follow up of only one month. However, because the other outcomes reported had a follow up of 1 year, ST reported in that particular trial were assessed along with the other trials having a follow up period of 12 months. However, the results were not affected. Another trial had a follow up period of 15 months. It was included in this analysis and was expected to partly compensate for the trial which had a follow up period of one month for ST. Furthermore, a high level of heterogeneity was observed in the subgroup analyzing subacute probable ST. Even if this was negligible since this high level of heterogeneity was present in only one subgroup, this could also contribute to the limitations observed in this study. The fact that different types of patients were included, for example a few studies involved only patients with diabetes mellitus, other studies involved patients with left main coronary artery disease and so on, this might have had an influence on the results obtained. In addition, it might be possible that the subgroup assessing ‘any definite ST’ was influenced by trials DUTCH PEER and HOST ASSURE. However, because ST with several different definitions and types were included in that particular subgroup, which might have been the cause for this difference, this particular result might be ignored. Another limitation could be the fact that this current analysis included patients who were implanted with non-resorbable polymer EES, which are older compared to recent EES with resorbable polymer which are nowadays being used.

## Conclusion

At 1 year follow up, ZES were not associated with significantly lower or higher definite and probable ST compared to EES. In addition, no significant difference was observed in acute, subacute and late definite or probable ST. However, further trials are recommended to assess the effects of these second-generation DES during the long-term.

## Abbreviations

DES: Drug eluting stents
EES: Everolimus eluting stents
PCI: Percutaneous coronary intervention
RCTs: Randomized controlled trials
ST: Stent thrombosis
ZES: Zotarolimus eluting stents
